# Paraplegia due to recurrent multiple hydatid cyst of the spine: A case report

**DOI:** 10.4103/0019-5413.40262

**Published:** 2008

**Authors:** Kshitij Chaudhary, Mihir Bapat, Siddharth Badve

**Affiliations:** Department of Orthopedics, King Edward Memorial Hospital, Mumbai, India

**Keywords:** Recurrent hydatid cyst, hydatid cyst of spine, radical excision

## Abstract

Recurrence after surgical treatment of hydatid cyst of the spine is extremely common. Preexisting fibrosis, fragility of the cyst wall, confluent cysts and proximity to vital structures makes radical excision difficult and repeated recurrences are inevitable. This case report describes a recurrent hydatid cyst presenting as three separate cysts in the dorsal spine in a middle-aged male. The extradural cyst caused paraplegia. The extraspinal cyst presented as an extrapleural mass in relation with the eighth, ninth and the tenth ribs near the costo-vertebral junction. The three cysts were resected en masse. Complete neurological recovery occurred with no recurrence at four years follow-up. Resection of the hydatid cyst en masse offers the best chance of cure and must be attempted in all cases. A prolonged chemotherapy should be administered in all cases.

Recurrent spinal hydatid disease causing cord compression is a rare occurrence. Multiple surgeries in such situations are associated with a high rate of mortality. Treatment of recurrent lesions is controversial. En masse excision of the lesion wherever possible, is the only way of achieving a lasting cure. The exact role of chemotherapy is unclear.

## CASE REPORT

**Figure 1:**
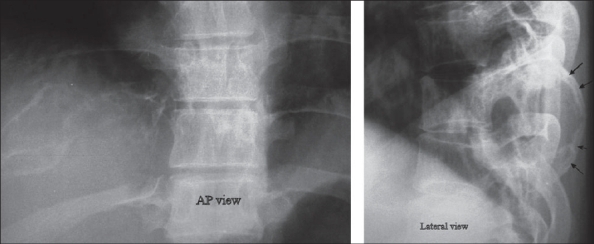
Preoperative radiographs showing cyst in the eighth rib. Notice that the normal outline of the right-sided eighth rib is not visible due to the expansile lytic lesion. Lateral X-ray shows the expansile lytic lesion (arrows)

**Figure 2:**
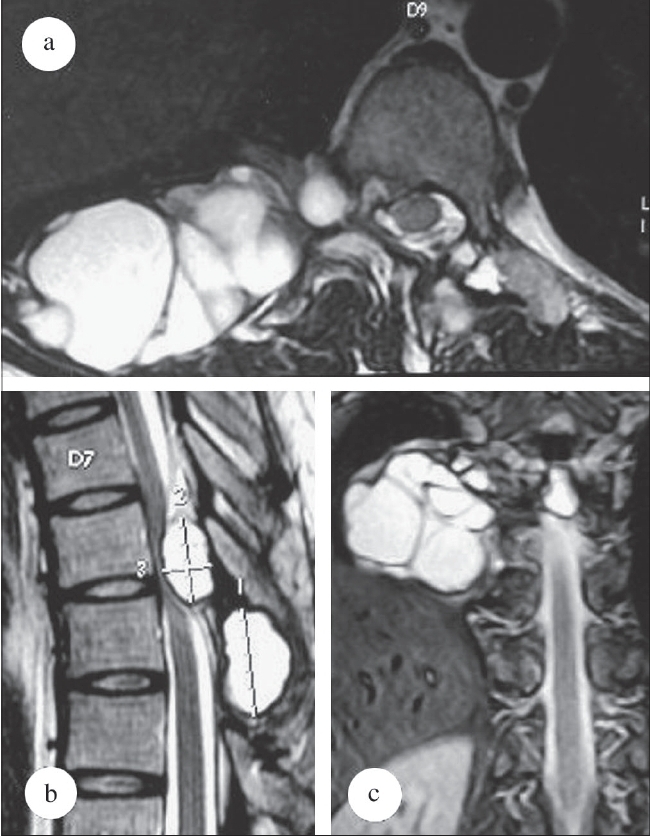
Preoperative MRI images T2 Axial (a) Sagittal (b) and coronal (c) images showing intraspinal and extraspinal cysts

A 48-year-old male presented with progressively worsening pain in the thoracic spine radiating along the right eighth rib of four weeks duration and spastic paraplegia with bladder involvement of two weeks duration. He had undergone intralesional curettage for a progressively enlarging mass in the right eighth rib two years ago. The patient had received anti-echinococcus chemotherapy for six months. The radiographs revealed a residual cyst in the eighth rib with intrathoracic extension [Figure 1]. The vertebral column was not involved. The MRI revealed three separate well-circumscribed cysts. The residual extraspinal intrathoracic cyst had enlarged on the under-surface of the rib cage extending from the lower border of the seventh to the upper border of the tenth rib [Figure 2]. The cyst measured 5 cm in diameter. The cyst was multiloculated and hyperintense on T2 weighted images with a hypointense well-demarcated cyst wall. It had involved the rib head but the pedicle of the T8 vertebra was uninvolved. Two unilocular cysts measuring 2 cm in diameter were located at the level of the T8 and T9 vertebrae. One of these cysts was intraspinal and extradural and produced compression of the spinal cord while the other cyst was located outside the spinal canal in relation to the lamina of the T9 vertebra [Figure 2B]. The eosinophil count, erythrocyte sedimentation rate and Casoni's test were within normal limits.

**Figure 3:**
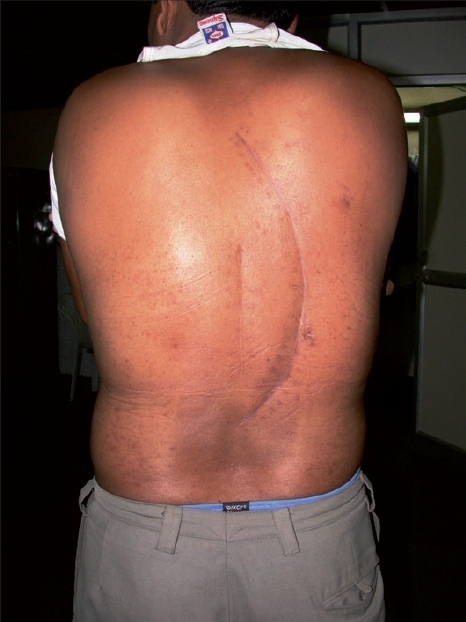
Postoperative clinical picture showing two scars. The midline vertical scar is of the first surgery, in which intralesional excision was done. The second curvilinear scar is of the second surgery in which en masse resection of the recurrent cyst was done.

**Figure 4:**
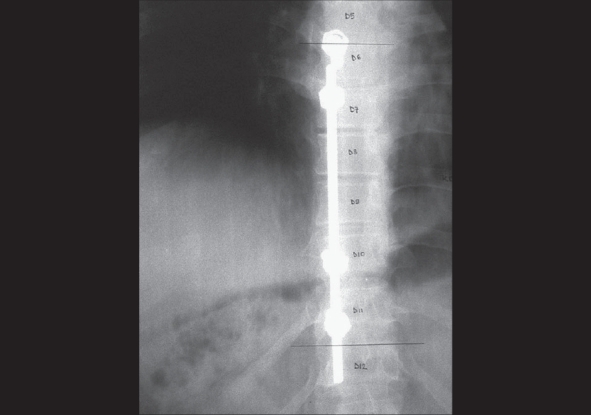
Four-year follow-up AP radiograph showing loosening of the rod from the screw at the upper end. The rod and screws were subsequently removed

Using a right-sided posterior paramedian curvilinear incision [Figure 3], a myocutaneous flap was raised towards the midline to expose the seventh to the tenth ribs from the posterior axillary line to their respective costo-vertebral junctions. The seventh, eighth, ninth and tenth ribs were extraperiosteally resected medially at the costotransverse junction and laterally beyond the lateral most extent of the cyst wall. The parietal pleura were incised at the periphery and chlorhexidine packs were placed inside the thoracic cavity to isolate the cyst. The cyst fluid was aspirated and 10 cc of hypertonic saline was injected into the cyst for its scolicidal effect. The cyst was gently separated from the lateral to the medial side by blunt dissection. The lateral part of the T8 pedicle was osteotomised along with the adjoining rib-head and the entire cyst excised. The T9 spinal cyst was excised en masse during T8 and T9 laminectomy. The T8 intraspinal cyst was gently lifted off the dura to achieve decompression of the thoracic cord. There was no communication between the intraspinal and extraspinal cysts. A single left ipsilateral rod and pedicle screw was used to stabilize the spine along with posterior bone grafting [Figure 4].

**Figure 5:**
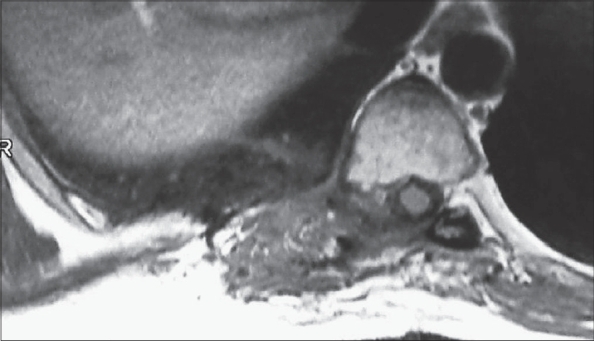
Postoperative T1 WI axial MRI showing resolution of cyst. The lamina and the right-sided pedicle and rib were surgically excised along with the cyst. There are no fluid-filled cystic areas. The size of the cord is restored

The patient reached full functional status by six months and received albendazole for 12 months. Rod loosening was noticed after four years which was removed subsequently. Alignment of the thoracic spine was maintained and did not show any signs of collapse on follow-up radiographs. Magnetic resonance imaging (MRI) done at four years showed resolution of the cyst [Figure 5].

## DISCUSSION

Spinal hydatidosis accounts for 50% of all musculoskeletal hydatid infections.^1^–^3^ Due to the rarity of presentation, a high index of suspicion is required in early diagnosis of these lesions. Classical radiological appearance of multilocular cystic disease of the bone with fluid levels is seen in 30% of the cases. The commonest differential diagnosis of early lesions is tuberculosis and a majority of these patients are inadvertently treated with anti-tubercular chemotherapy.^2^,^3^ Spillage of the infectious cyst contents during a biopsy results in aggressive and widespread recurrences. In diagnosed cases, radical resection of the mother cyst is the procedure of choice.^4^,^5^ En masse excision of the spinal lesion depends largely on the location and the extent of the lesion. In infiltrative anterior osseous lesions, the fragility of the cyst wall and the penetration of microvesicles into the substance of the vertebral body result in penetration of the vessel wall during surgery. Small well-circumscribed anterior, epidural or intradural cysts are often amenable to radical excision.^6^–^8^

Pain results from a compressive radiculopathy or due to erosion of the vertebral corpus. Spinal compression causing slowly progressive myelopathy is seen in three situations in decreasing order of frequency: 1. Contiguous spread of an intrathoracic or a retropleural cyst through the neural foramen into the spinal canal. 2. Epidural spread of an anterior osseous cyst and 3. A discreet epidural or an intradural cyst.^6^–^8^,^9^,^10^ In the present case, three separate cysts were identified and were radically excised. In the former two situations radical resection is extremely difficult and more often than not intralesional debridement becomes the procedure of choice to achieve neural decompression. Subsequent repetitive intralesional curettage seldom provides satisfactory neurological recovery and the morbidity arising therein results in mortality in 50% of the cases after an average of five surgical procedures.^8^,^9^ Visceral dissemination most commonly to the liver occurs in 10% of the patients after surgery for recurrent cysts. Injection of hypertonic saline and isolation of the surgical wound with chlorhexidine packs are important adjuncts; however, following a cyst rupture, the usefulness of these scolicidal agents is doubtful and recurrences are common.

The three most widely used drugs are albendazole, mebendazole and praziquantel. Effectiveness of these agents, both in terms of the dose and duration of treatment and role of combination therapy is controversial. Albendazole has better intestinal absorption, a longer half life of 8.5 h and a higher cyst concentration than the other drugs.^11^–^13^

It is unclear whether lesions that may be amenable for radical surgery should be treated medically. Benign slow-growing asymptomatic lesions are best treated medically rather than risking an inadvertent surgical rupture, unless these lesions are in close proximity to the spinal cord. In recurrent infiltrative lesions, it is probably wise to combine protracted chemotherapy with punctuated palliative surgeries aimed to improve the quality of life of the patient.

There are no criteria on MRI to differentiate between an infectious and a non-infectious treated cyst if the latter does not completely resolve.^14^ It is unclear whether a treated cyst demonstrates a late recurrence. Therefore, precise guidelines as to when treatment can be discontinued remain controversial.^12^
